# Self-supervised learning analysis of multi-FISH labeled cell-type map in thick brain slices

**DOI:** 10.3389/fnins.2025.1622950

**Published:** 2025-07-07

**Authors:** Weijie Zheng, Yiping An, Kang Li, Jinyue Wang, Jianqing Gao, Huawei Mu, Jin Tang, Hao Wang

**Affiliations:** ^1^AHU-IAI AI Joint Laboratory, Anhui University, Hefei, China; ^2^Anhui Province Key Laboratory of Biomedical Imaging and Intelligent Processing, Institute of Artificial Intelligence, Hefei Comprehensive National Science Center, Hefei, China; ^3^Institute of Advanced Technology, University of Science and Technology of China, Hefei, China; ^4^MoE Key Laboratory of Brain-inspired Intelligent Perception and Cognition, National Engineering Laboratory for Brain-inspired Intelligence Technology and Application, School of Information Science and Technology, University of Science and Technology of China, Hefei, China; ^5^iFlytek Research, iFlytek Co., Hefei, China; ^6^Hefei National Research Center for Physical Sciences at the Microscale, School of Life Sciences, Division of Life Sciences and Medicine, University of Science and Technology of China, Hefei, China

**Keywords:** cell type atlas, cell segmentation, self-supervised learning, fluorescence *in situ* hybridization, light sheet microscopy

## Abstract

**Introduction:**

Accurate mapping of the spatial distribution of diverse cell types is essential for understanding the cellular organization of brain. However, the cellular heterogeneity and the substantial cost of manual annotation of cells in volumetric images hinder existing neural networks from achieving high-precision segmentation of multiple cell-types within a unified framework.

**Methods:**

To address this challenge, we introduce a self-supervised learning framework, Voxelwise U-shaped Swin-Mamba network (VUSMamba), for automatic segmentation of multiple neuronal populations in 300 μm thick brain slices. VUSMamba employs contrastive learning and pretext tasks for self-supervised learning on unlabeled data, followed by fine-tuning with minimal annotations. As a proof of concept, we applied the framework to a multi-cell-type dataset obtained using multiplexed fluorescence in situ hybridization (multi-FISH) combined with high-speed volumetric microscopy VISoR.

**Results:**

Compared to state-of-the-art baseline models, VUSMamba achieves higher segmentation accuracy with reduced computational cost. The framework enables simultaneous high-precision segmentation of glutamatergic neurons, GABAergic neurons, and nuclei.

**Discussion:**

This work presents a unified self-supervised neural network framework that offers a standardized pipeline for constructing and analyzing whole-brain cell-type atlases.

## 1 Introduction

Cells within organisms can be classified into distinct types based on shared structural and functional characteristics, facilitating the study of cellular organization and functional heterogeneity (Arendt, 2008). As the central organ regulating bodily functions, the brain necessitates a comprehensive cell type atlas to elucidate the roles and interactions of diverse cell populations across regions. Recent advances in fluorescence *in situ* hybridization (FISH), single-cell sequencing, and spatial transcriptomics have greatly enhanced our ability to profile gene expression and cellular functions. FISH visualizes target RNA or DNA molecules via fluorescent probes (Choi et al., 2018; Femino et al., 1998), while single-cell sequencing reveals transcriptomic heterogeneity at the individual cell level (Armand et al., 2021; Tanay and Sebé-Pedrós, 2021). Spatial transcriptomics integrates sequencing with spatial information, preserving tissue context during gene expression analysis (Zhuang, 2021; Rao et al., 2021). These technologies have enabled the construction of region-specific brain cell atlases, providing critical insights into the cellular architecture and function of the mammalian brain.

Recent efforts have integrated single-cell RNA sequencing with spatial transcriptomics to construct cell type atlases of specific mouse brain regions. The MERFISH enabled the generation of a spatially resolved molecular atlas for the primary motor cortex and adjacent areas (Zhang et al., 2021), and was later extended to image over 1,100 genes in 8 million cells across the entire adult mouse brain, identifying over 5,000 transcriptionally distinct cell clusters (Zhang et al., 2023). This whole-brain atlas was built by integrating single-cell (~ 7 million cells) and spatial transcriptomic (~ 4.3 million cells) datasets acquired via MERFISH. However, these atlases relied on sparsely sampled 10 μm slices, limiting their comprehensiveness (Chen et al., 2015; Shi et al., 2023). To overcome this, methods such as STARmap enabled profiling in 150 μm tissue blocks (Wang et al., 2018), while EASI-FISH further extended thickness to 300 μm, allowing detailed molecular analysis in the lateral hypothalamic area (LHA) (Wang et al., 2021). Recent advances in MERFISH protocols have achieved 3D imaging of 200 μm-thick slices, enhancing speed and accuracy via deep learning (Fang et al., 2024).

Building upon these developments, this study employs modified hybridization chain reaction (HCR) (Choi et al., 2018) FISH labeling combined with the Volumetric Imaging with Synchronized on-the-fly-scan and Readout (VISoR) (Wang et al., 2019) high-speed volumetric imaging system to obtain multi-FISH labeled cell type map data from continuous 300 μm thickness slices. To enable accurate segmentation of multiple cell types, deep learning models typically require extensive expert annotations. Self-supervised learning offers an alternative by deriving supervisory signals from unlabeled data, such as predicting spatial context or reconstructing missing regions.

Self-supervised learning (SSL) leverages the intrinsic structure of data to generate supervisory signals, enabling model training without manual annotations. Through pretext tasks, SSL models learn transferable representations applicable to downstream tasks such as classification, detection, and segmentation. Contrastive learning is a prominent SSL approach, exemplified by methods such as InstDisc and MoCo, which construct positive and negative pairs to enhance feature consistency (Wu et al., 2018; He et al., 2020). The SimCLR further streamlined this framework using data augmentation to generate positive pairs, marking a significant milestone in computer vision (Chen et al., 2020). In biomedical research, SSL has shown transformative potential. AlphaFold2 leveraged SSL for accurate protein structure prediction, revolutionizing protein folding studies (Jumper et al., 2021). Given the high annotation cost in biomedical imaging, SSL has been widely adopted in segmentation tasks. For instance, SwinUNETR integrates a hierarchical Transformer encoder with SSL-based pre-training, achieving strong performance across multiple medical segmentation benchmarks (Tang et al., 2022). Despite their effectiveness, Transformer-based models often suffer from high computational complexity, limiting deployment in resource-constrained settings. Mamba, a recent architecture based on state space models (SSMs), addresses this by introducing selective state space transitions for efficient long-sequence modeling with linear complexity (Gu et al., 2021). The Mamba has been successfully applied to biomedical image segmentation, reducing computational demands while outperforming based on convolutional neural network (CNN) and transformer architectures (Liu et al., 2024a,b). Its efficiency makes it particularly suitable for large-scale whole-brain neuronal image datasets, facilitating high-throughput single-cell segmentation with reduced hardware requirements.

Therefore, we propose a novel self-supervised neural network architecture, Voxel-wise U-shape Swin-Mamba Network (VUSMamba), for end-to-end segmentation of Hoechst-, *Vglut1*-, and *Vgat*-positive cells in thick brain slices. The workflow begins with preprocessing of the image data for the three types of labeled cells (Hoechst, *Vglut1, Vgat*), followed by the construction of a self-supervised training dataset. Hoechst staining was used to label cell nuclei, serving as a reference for the localization of other fluorescent signals. Three pretext tasks—rotation prediction, image reconstruction, and image recovery—are designed to enable representation learning through contrastive self-supervised learning. The pre-trained model is then fine-tuned using a small set of manually annotated ground truth (GT) data. Finally, we quantify the densities of *Vglut*1^+^, *Vgat*^+^, and co-expressing *Vglut*1^+^-*Vgat*^+^ cells across multiple brain regions. Based on the spatial distribution patterns of *Vglut*1^+^ and *Vgat*^+^ cells within selected regions, boundary lines are computed and compared with anatomical boundaries defined by the Allen mouse brain atlas Common Coordinate Framework (CCFv3) (Wang et al., 2020).

## 2 Materials and methods

**Figure 1:**
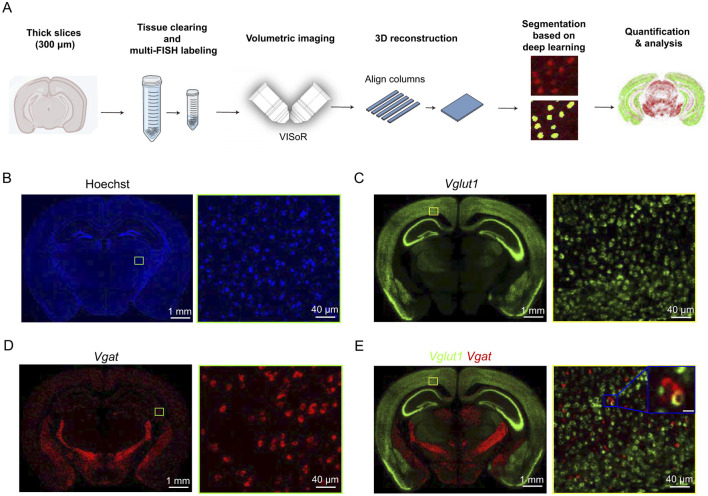
Diagram of this study. **(A)** Schematic of the experimental workflow. Mouse brain tissues were sectioned into thick slices (300 μm), followed by tissue clearing and FISH labeling. Samples were imaged using the VISoR system, aligned and reconstructed into brain slice image. Deep learning-based segmentation was used for automatic cell identification and quantification of gene expression across the brain. **(B–D)** Representative coronal images [Hoechst **(B)**, *Vglut1* **(C)**, *Vgat* **(D)**] showing nuclear distribution and gene expression at both the brain slice (left) and single-cell resolution (right). **(E)** Merged image showing co-localization of *Vglut1* and *Vgat* signals. Scale bar: 10 μm. The thickness of the maximum intensity projection for the images is 2 μm.

The overall workflow of this study consisted of six major steps: brain slice embedding, tissue clearing and FISH labeling, high-speed volumetric imaging using VISoR, 3D reconstruction of brain slices, deep learning-based cell segmentation, and quantitative analysis (Figure 1A).

### 2.1 Sample preparation

C57BL/6 or virus-injected mice were deeply anesthetized with 1% (w/v) sodium pentobarbital. Cardiac perfusion was performed sequentially with 20 mL of 37°C 1 × PBS (phosphate buffered saline; dissolved in RNase-free water), 20 mL of ice-cold 1 × PBS, and 20 mL of ice-cold 1% hydrogel monomer solution (HMS; 1% acrylamide, 0.0125% bis-acrylamide, 0.25% VA-044 initiator [w/v], 4% PFA in 1 × PBS, RNase-free) (Sylwestrak et al., 2016). Brains were dissected and incubated in 40 mL 4% HMS at 4°C overnight. The embedding solution (20 ml of 4% HMS and 20 ml of 4% BSA) was degassed under vacuum for 10 min. Brains were then immersed in the solution and sealed for polymerization at 37°C for 4 h. Embedded brains were trimmed and sectioned into 300 μm slices using a vibratome (B-S-1018, Bitelligen). Slices were cleared overnight at 37°C in 4% SDS/0.2 M boric acid buffer (pH = 8.5) with gentle shaking (Supplementary Figure 1), then washed three times in 0.3% PBST (1 × PBS with 0.3% Triton X-100) at 37°C for 1 h each, followed by a final wash in 1 × PBS at room temperature.

### 2.2 Fluorescence *in situ* hybridization

The hybridization protocol was adapted from the HCR 3.0 method (Choi et al., 2018). Mouse brain slices were transferred to 5 mL tubes and incubated in 1 mL of 30% pre-hybridization buffer (30% formamide in 5 × SSC) at 37°C for 30 min with gentle shaking. Slices were then incubated overnight at 37°C in 1 mL of probe hybridization buffer (30% formamide in 5 × SSC containing a probe mixture; 400 nM per probe) with gentle shaking. The following day, slices were washed at 37°C with 30% pre-hybridization buffer four times (2 × 15 min, then 2 × 30 min) with gentle shaking, followed by two washes at room temperature in 5 × SSCTw (5 × SSC with 0.1% Tween-20) for 15 min each. Samples were then equilibrated in pre-amplification buffer (5 × SSCTw) at room temperature for 30 min. Fluorescent hairpins were prepared by snap-cooling 20 μL of 3 μM hairpin stock in hairpin buffer (heated at 95°C for 90 s, then cooled in the dark at room temperature for 30 min). The snap-cooled hairpins were added to 1 mL of amplification buffer. Samples were incubated overnight (>16 h) at room temperature in the dark with 1 mL of amplification buffer. Excess hairpins were removed by washing at room temperature in 5 × SSCTw (2 × 5 min, 2 × 30 min), 0.5 × SSCTw (2 × 30 min), and 0.5 × SSC (3 × 10 min) with gentle shaking.

### 2.3 Imaging

Prior to imaging, brain slices were incubated overnight in a refractive index (RI) matching medium composed of iohexol (650 g), urea (350 g), triethanolamine (140 g), and 210 mL of RNase-free water. The imaging chamber was filled with the same medium to ensure consistent optical properties during acquisition. Imaging was conducted using the VISoR platform (Wang et al., 2019; Xu et al., 2021), equipped with four excitation lasers (405, 488, 561, and 647 nm; Coherent OBIS series) and a Hamamatsu Flash 4.0 v3 sCMOS camera. Image acquisition was performed with a 10 ×, 0.3 numerical aperture water-immersion objective (Olympus) and a 0.63 × relay lens (TV0.63, Olympus), yielding a final voxel resolution of 1 × 1 × 3.5 μm^3^.

### 2.4 Data preparation

Using the above protocol, Hoechst, *Vglut1*, and *Vgat* signals were labeled. VISoR imaging followed by 3D reconstruction (Supplementary material) enabled visualization of different cell types (Figures 1B–E and Supplementary Video 1). The self-supervised training dataset consists of volumetric images labeled with Hoechst, *Vglut1*, and *Vgat* signals, each with a size of 64 × 256 × 256 pixels and a voxel resolution of 2 × 2 × 2 μm^3^. A total of 13,736 volumes were included. Each reconstructed 3D brain slice (64 × 7, 000 × 5, 000 pixels) was divided into 320 sub-volumes of 64 × 256 × 256 pixels. For transfer learning, an additional dataset of 640 expert-annotated volumes was used (Supplementary Table 1). Due to the high density of cells expressing *Vglut1* and *Vgat* in the midbrain, the fine-tuning dataset was primarily constructed by selecting multiple sub-volumes from this region containing Hoechst, *Vglut1*, and *Vgat* signals.

### 2.5 Self-supervised learning

The sub-volume datasets are normalized before being inputted into the neural network for training. The normalization formula is:


(1) $$\begin{array}{l} N\left(x\right)=\frac{x-\min}{\max-\min} \end{array}$$

*max* and *min*, which are hyperparameters, are set to 112 and 1,000, respectively.

The training and testing of the network were completed on a workstation equipped with an NVIDIA GeForce RTX 3090 with 24 GB RAM. The neural networks implemented based on Python 3.10 and Pytorch 2.2.1. The network training utilized the AdamW optimizer, with a parameter learning rate of 1 × 10^−3^ and a weight decay rate of 0.1. The warmup cosine learning rate schedule was employed to promote stable training and smooth convergence. We employed contrastive learning in self-supervised training to enable the neural network to learn high-dimensional representation information. The network was trained for 100 epochs with a batch size of 1. Self-supervised learning typically leverages context reconstruction and contrastive encoding to capture representative features of images (Haghighi et al., 2021; Zhou et al., 2021; He et al., 2020). Inspired by previous work, we designed three proxy tasks (rotation task, contrastive task and recovery task) to facilitate representation learning for fluorescence microscopy images. Specifically, the rotation task helps the model capture the structural characteristics of 3D images and produces diverse sub-volumes for use in contrastive learning. The contrastive task enables the model to differentiate between regions of interest (ROIs) associated with distinct cell types, while the recovery task allows it to learn the contextual associations between various structures and their surrounding environments. The loss function is composed of three parts: rotation loss, contrastive loss, and recovery loss. Their computation formulas are as follows:


(2) $$l_{rot}=-\sum_{r=1}^{R}y_{r}\log\left({\tilde{y}}_{r}\right)$$

(3) $$\begin{array}{l} l_{contrast}=-\log\frac{\exp\left(sim\left(v_{i},v_{j}\right)/t\right)}{\overset{2N}{\underset{k}{\sum }}1_{k\neq i}\exp\left(sim\left(v_{i},v_{k}\right)/t\right)} \end{array}$$

(4) $$\begin{array}{l} l_{recovery}={\left(Y_{gt}-Y_{out}\right)}^{2} \end{array}$$

In rotation prediction tasks, we designated rotations of 0°, 90°, 180°, and 270° along the z-axis as representatives of the R class. We employed Equation 2 to calculate their cross-entropy loss, where *y*^*r*^ and ỹ^*r*^ represented the probability values of the true class and the predicted rotation class, respectively.

Contrastive encoding demonstrated superior capability in learning visual representation information within self-supervised learning (Chen et al., 2020; Park et al., 2020). The implementation of contrastive encoding involved adding a linear mapping layer after the encoding layer of the neural network to maximize the mutual information between positive samples (sub-volumes from the same volumetric image) and minimize the mutual information between negative samples (sub-volumes from different volumetric images) in the output. The Equation 3 was employed to compute the contrastive loss between the mentioned samples. Here, *sim*(·) represented the cosine similarity function, while *v*_*i*_, *v*_*j*_, and *v*_*k*_ stand for the latent representations of different samples. The parameter *t* denoted the normalized temperature value. The indicator function 1 was utilized to be 1 when *k* is not equal to *j*. *N* denoted the total number of samples in the self-supervised training dataset.

**Figure 2:**
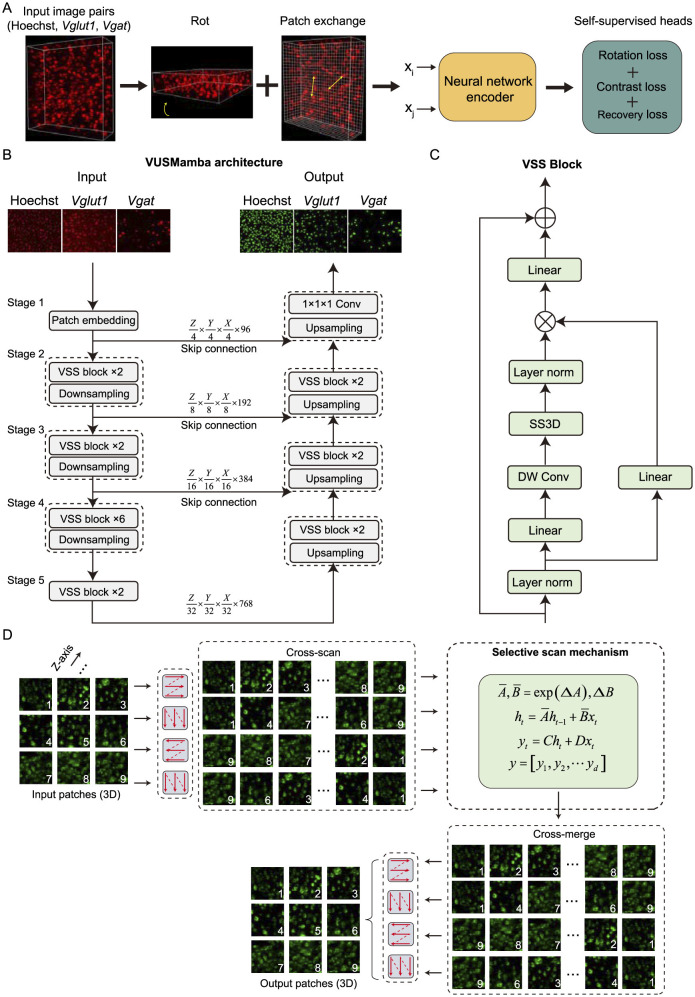
Self-supervised learning details and VUSMamba network structure. **(A)** A flowchart of the self-supervised training process. The *x*_*i*_ and *x*_*j*_represent the two input contrastive images, and contrastive learning is performed through image rotation prediction, recovery, and reconstruction. The *l*_*rot*_, *l*_*contrast*_, and *l*_*recovery*_ correspond to the loss functions for the different operations mentioned above. The yellow arrows point to the results after patch exchange. **(B)** A flowchart of VUSMamba for end-to-end 3D cell segmentation. **(C)** Details of the VSS block composition and tensor processing workflow. **(D)** A flowchart of the SS3D for extracting image features.

In the task of volumetric image recovery, we developed a method called context-aware volumetric patch exchange for image recovery. In particular, we randomly sampled volumetric patches of size 30 × 30 × 30 from the input volume image for random exchange. The exchanged image then underwent a neural network encoding-decoding structure for image restoration, with the resulting mean squared error (MSE) loss computed against the original image. The Equation 4 was used to compute the MSE loss between them, where *Y*_*gt*_ and *Y*_*out*_ represent the original image and the predicted image recovery result, respectively. The three aforementioned loss functions collectively form the loss function for self-supervised training:


(5) $$\begin{array}{l} l=\lambda_{1}l_{rot}+\lambda_{2}l_{contrast}+\lambda_{3}l_{recovery} \end{array}$$

where λ_1_, λ_2_ and λ_3_ are hyperparameters (Figure 2A). The ablation study (Supplementary Table 2) demonstrated that the model achieved optimal segmentation performance when the hyperparameters were set to λ_1_ = λ_2_ = λ_3_ = 1.

### 2.6 VUSMamba framework

In this section, we provide an elaborate exposition on the VUSMamba neural network, with Mamba serving as its central component. The VUSMamba framework consisted of two main parts: encoder and decoder, presenting an overall U-shaped architecture (Figure 2B). The encoder primarily comprised a Patch embedding layer, Visual State Space (VSS) blocks (Figure 2C), and Patch merging layer. The VSS blocks were stacked with parameters [2, 2, 6, 2]. The decoder was primarily composed of VSS blocks, Patch expanding layers, and a convolutional layer with a kernel size of 1 × 1 × 1. The VSS blocks were stacked with parameters [2, 2, 2]. The skip connections in the VUSMamba network enabled the decoder to combine multi-scale features obtained from the encoder during the up-sampling process, thereby enhancing the accuracy of cell segmentation. The following provided a detailed description of the structure of each layer and module.

#### 2.6.1 Patch embedding layer

This layer was primarily composed of 3D convolutional kernels and LayerNorm (LN) layers. The input image (*W*×*H*×*D*×1) was divided into multiple non-overlapping tokens ($\frac{W}{4}\times \frac{H}{4}\times \frac{D}{4}\times C$), and the channel dimension is mapped to a high-dimensional space (defined as *C*). The LN layer can normalize data along the channel dimension.

#### 2.6.2 VSS block

This block mainly consisted of four components: LN layer, linear mapping layer, depth wise convolution (DW Conv), and 3D selective scan (SS3D). The core part of the block was SS3D, which employed a discretized selective scan mechanism (S6) (Gu et al., 2021) to extract three-dimensional image features (Figure 2D). The input image was separated into multiple tokens through the Patch embedding layer, forming a tensor of length *L*, denoted as $v_{k}\in {\mathbb{R}}^{L\times C}$. Then, *v*_*k*_ sequentially passed through the LN layer, linear mapping layer, and DW Conv, before being passed to SS3D for feature extraction. The calculation method after discretization using the zeroth-order hold rule for the ordinary differential equations (ODEs) used in feature extraction is as follows:


(6) $$h_{k}=\bar{A}h_{k-1}+\bar{B}v_{k}$$

(7) $$\begin{array}{l} y_{k}=Ch_{k}+Dv_{k} \end{array}$$

(8) $$\begin{array}{l} \bar{A}=e^{\Delta A} \end{array}$$

(9) $$\begin{array}{l} \bar{B}=\left(e^{\Delta A}-1\right)A^{-1}B \end{array}$$

where *A* ∈ ℝ^*N*×*N*^, *B, C* ∈ ℝ^*N*×*L*^, Δ ∈ ℝ^*N*×*N*^, *D* ∈ ℝ^1^ and *N* was a state size. In Equation 9, $\bar{B}$ was approximated using the first-order Taylor series expansion:


(10) $$\begin{array}{l} \bar{B}\approx \left(\Delta A\right){\left(\Delta A\right)}^{-1}\Delta B=\Delta B \end{array}$$

In a 2D image, the image was unfolded into a sequence along rows and columns, and information from other pixels is obtained by scanning in four different directions (Liu et al., 2024). Similarly, we unfolded the image into a sequence along depth, rows, and columns, and still scan in four different directions to obtain information from other pixels in 3D images (Figure 2C). Assuming the input features for SS3D are denoted as z, the calculation process described above is as follows:


(11) $$\begin{array}{l} z_{d}=expand\left(z,d\right) \end{array}$$

(12) $$\begin{array}{l} {\tilde{z}}_{d}=S6\left(z_{d}\right) \end{array}$$

(13) $$\begin{array}{l} \tilde{z}=merge\left({\tilde{z}}_{1},{\tilde{z}}_{2},{\tilde{z}}_{3},{\tilde{z}}_{4}\right) \end{array}$$

where $\tilde{z}$ is an output feature of SS3D and *d* ∈ {1, 2, 3, 4} is four different directions.

**Patch merging layer** and **patch expanding layer**. These two layers could respectively perform 2 × down-sampling and up-sampling on the feature sequence, with the channel dimension increasing and decreasing by a factor of 2 correspondingly. Assuming the input feature shape to the Patch merging layer was $\frac{W}{4}\times \frac{H}{4}\times \frac{D}{4}\times C$, then the output feature shape was $\frac{W}{8}\times \frac{H}{8}\times \frac{D}{8}\times 2C$. Likewise, assuming the input feature shape to the Patch expanding layer was $\frac{W}{32}\times \frac{H}{32}\times \frac{D}{32}\times 8C$, then the output feature shape was $\frac{W}{16}\times \frac{H}{16}\times \frac{D}{16}\times 4C$. Since the input image, after being processed by the patch embedding layer, became $\frac{1}{4}$ of its original size, the up-sampling factor for the final Patch expanding layer was 4.

**Skip connection** and **1 × 1 × 1** **conv**. The functions of these two structures were respectively to fuse multiple scale features to increase segmentation accuracy and to perform linear classification on up-sampled feature maps in the channel dimension.

### 2.7 Voxel-wise evaluation metrics

The voxel-wise evaluation metrics, including dice score (DSC), sensitivity (Sst), and jaccard coefficient (Jc), are used to evaluate the performance of neural network segmentation. The DSC calculated the Dice coefficient, measuring the overlap between the predicted segmentation results and the ground truth. It was calculated as:


(14) $$\begin{array}{l} \text{DSC}=\frac{2\text{TP}}{2\text{TP}+\text{FP}+\text{FN}} \end{array}$$

where true positives (TP) were the number of correctly predicted positive pixels, false positives (FP) were the number of incorrectly predicted positive pixels and false negatives (FN) were the number of incorrectly predicted negative pixels. The Jc evaluated the similarity between the predicted segmentation set and the ground truth set. The Sst assessed the proportion of positive targets in the predicted set relative to the positive targets in the ground truth set. Their calculation formulas were as follows:


(15) $$\begin{array}{l} \text{Jc}=\frac{\text{TP}}{\text{TP}+\text{FP}+\text{FN}} \end{array}$$

(16) $$\begin{array}{l} \text{Sst}=\frac{\text{TP}}{\text{TP}+\text{FN}} \end{array}$$

In addition to the fact that a higher values of Dice, Jc, and Sst indicated better segmentation performance.

### 2.8 Cell-type detection

After segmentation by VUSMamba, the Hoechst images were processed using a connected component analysis method to identify individual cell nuclei and extract their centroid coordinates and volumes. To ensure reliable segmentation, nuclei with volumes between 110 μm^3^ and 540 μm^3^ were considered valid. For cell type detection, we determined gene expression based on the spatial correspondence between Hoechst-labeled nuclei and other marker signals. Specifically, if the segmented mask of a Hoechst-labeled cell overlapped with a non-zero mask in the segmentation results of another marker signal, the cell was considered positive for that gene; otherwise, it was considered negative. The detection of cell-type in the aforementioned cells was expressed using the following formula:


(17) $$G\left(x_{seg},y_{seg},z_{seg}\right)=\{\begin{array}{c} \begin{array}{cc} \text{True} & i\cap mask\neq \emptyset \end{array}\\ \begin{array}{cc} \text{False} & i\cap mask=\emptyset \end{array} \end{array}$$

(18) $$\begin{array}{l} i=I\left(x_{seg},y_{seg},z_{seg}\right) \end{array}$$

where *G*(·) was the gene expression matrix, *x*_*seg*_, *y*_*seg*_ and *z*_*seg*_ were the coordinates of the Hoechst image segmentation results. The *I*(·) was the image under different labeled signal. True and False indicated whether a cell expressed a particular gene.

## 3 Result

### 3.1 Comparison of segmentation results

In this study, VUSMamba was evaluated against state-of-the-art baseline models based on CNNs and transformer architectures (Ronneberger et al., 2015; Tang et al., 2022; Chen et al., 2024). All models were first pre-trained on a self-supervised dataset, and subsequently fine-tuned using a dataset annotated with ground truth (GT) labels. For the self-supervised training of SwinUNETR, proxy tasks were used in accordance with its original design, without any modifications. For baseline models that do not incorporate a built-in self-supervised training strategy, the proxy tasks proposed in this study were applied to ensure consistent evaluation of all models under self-supervised pre-training. The test set consisted of single brain slice data from independent samples (Supplementary Table 1). For each cell type, the slices were partitioned into 320 sub-volumes, which were manually annotated and cross-validated by multiple expert reviewers.

The comparative methods include U-Net, a CNN widely adopted in biomedical image segmentation; SwinUNETR, which demonstrates strong performance in medical image segmentation by employing customized pretext tasks and has achieved outstanding results across multiple public datasets; and CP-Net, which utilizes a hierarchical segmentation strategy from global to local regions, enabling effective segmentation of fine subcellular structures within cells. 3D-HSFormer is a neural network architecture designed for efficient and high-precision whole-brain c-Fos^+^ cell segmentation through supervised learning (Zheng et al., 2024).

**Figure 3:**
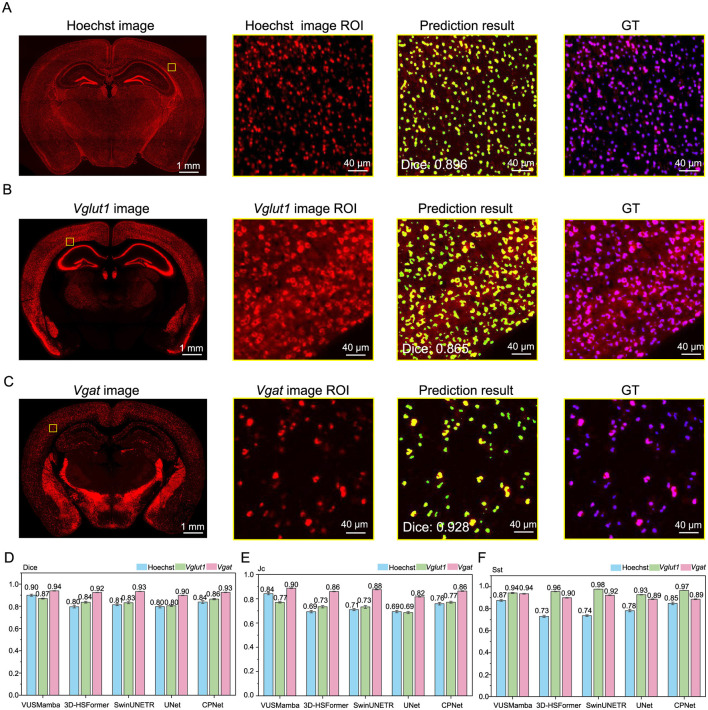
VUSMamba segmentation results are shown and compared with the baseline model. **(A–C)** Representative examples of segmentation results for nuclei and neurons labeled with different markers [Hoechst **(A)**, *Vglut1* **(B)**, *Vgat* **(C)**] in mouse brain slices. The thickness of the maximum intensity projection for the images is 2 μm. **(D–F)** Quantitative evaluation of segmentation performance across different models using DSC **(D)**, Jc **(E)**, and Sst **(F)**.

The VUSMamba achieved high-precision segmentation results on brain slice data labeled with Hoechst, *Vglut1*, and *Vgat* (Figures 3A–C, Supplementary Figure 2 and Supplementary Video 2). The multi-FISH labeling signals for both *Vglut1* and *Vgat* exhibited uniform intensity and spatial distribution across different z positions, confirming consistent staining quality throughout the imaging depth. Correspondingly, the segmentation performance of VUSMamba remained stable across z positions, as reflected by consistently high Dice coefficients, demonstrating its robustness to depth-dependent signal variations (Supplementary Figure 3). The VUSMamba model demonstrated superior performance in segmenting Hoechst, *Vglut1*, and *Vgat* signals, outperforming other models in both DSC and Jc (Figures 3D, E). However, on the Sst metric, its performance was slightly lower than that of CP-Net (Figure 3F). This can be attributed to CP-Net's coarse-to-fine segmentation strategy, which offers a distinct advantage in detecting small cellular targets. Despite this, VUSMamba achieved high segmentation accuracy for nuclei while also offering several practical advantages, including low computational cost, stable convergence, and fast inference speed, making it highly competitive for real-world applications (Supplementary Table 3). Overall, both VUSMamba and CP-Net exhibited balanced and robust performance across diverse marker types. SwinUNETR, while excelling in certain metrics, showed suboptimal results for some categories, suggesting it may be more appropriate for specific tasks or require further tuning. Traditional models like UNet and 3D-HSFormer remained strong in select tasks but were generally outperformed in overall capability.

Based on the segmentation performance of VUSMamba, it was applied to segment Hoechst, *Vglut1*, and *Vgat* signals on three consecutive brain sections from three adult mice. VUSMamba achieved high scores across all three segmentation evaluation metrics in samples from the three different animals (Supplementary Figure 4).

### 3.2 Spatial heterogeneity in the distribution of glutamatergic and GABAergic neurons

**Figure 4:**
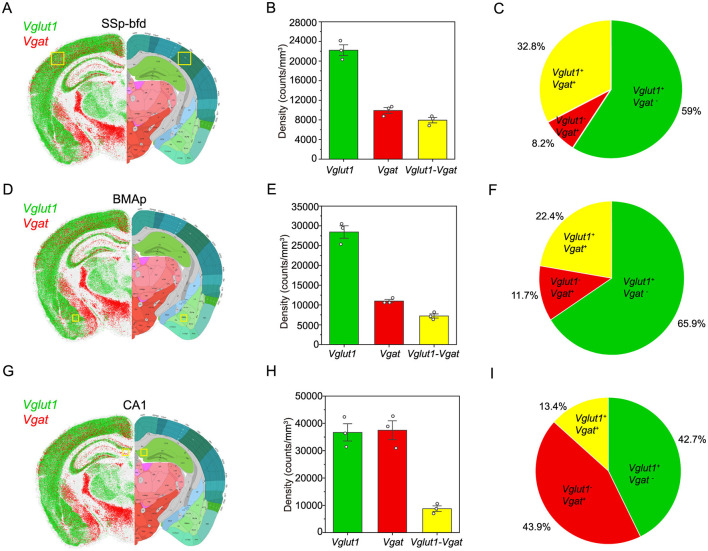
Spatial distribution and quantification of excitatory and inhibitory neurons in mouse brain areas. **(A, D, G)** Left: spatial localization of *Vglut1* (green) and *Vgat* (red) expressing cells in a coronal brain slice. Right: Allen mouse brain atlas overlay highlights anatomical boundaries; yellow boxes mark the quantified area (SSp-bfd, BMAp, CA1). **(B, E, H)** Bar graph showing the density of *Vglut*1^+^, *Vgat*^+^, and double-positive (*Vglut1*-*Vgat*) cells in the SSp-bfd (BMAp, CA1) area (n = 3). **(C, F, I)** Pie chart indicating the relative proportion of the three cell populations in the SSp-bfd (BMAp, CA1) area.

Understanding the cellular composition of different brain areas is fundamental to elucidating the mechanisms underlying functional specialization in the mammalian brain. Excitatory and inhibitory neurons, typically marked by the expression of *Vglut1* and *Vgat*, respectively, play distinct yet complementary roles in shaping regional neural circuits. To investigate the spatial distribution and relative abundance of these neuronal populations, three anatomically and functionally distinct areas of the mouse brain were examined: the primary somatosensory cortex barrel field (SSp-bfd), the hippocampal CA1 subfield, and the posterior part of the basomedial amygdalar nucleus (BMAp). Results demonstrate that the SSp-bfd (Figures 4A–C) and BMAp (Figures 4D–F) areas are predominantly composed of excitatory neurons, with *Vglut*1^+^ cells accounting for 59.0% and 65.9% of the total population, respectively. In contrast, the CA1 area (Figures 4G–I) exhibits a more balanced neuronal profile, with nearly equal proportions of *Vglut*1^+^ (42.7%) and *Vgat*^+^ (43.9%) cells.

In summary, the results reveal regional differences in neuronal composition across brain areas. SSp-bfd and BMAp are predominantly composed of excitatory neurons, whereas CA1 exhibits a relatively balanced distribution of excitatory and inhibitory neurons. These findings provide insight into the structural heterogeneity of neuronal networks in distinct functional areas of the brain and offer a foundational framework for future investigations into region-specific neural circuits and their associated functions.

### 3.3 Comparison between molecularly defined subregion boundaries and CCFv3 annotations

**Figure 5:**
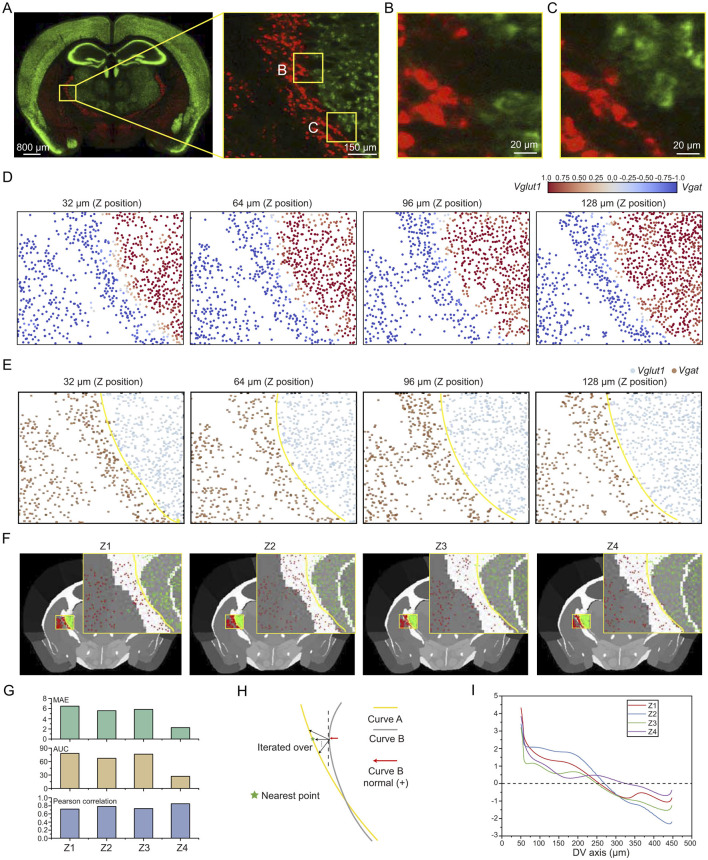
Molecularly defined brain region boundaries. **(A)** Left: Whole-brain coronal section showing the anatomical location of the analyzed region (yellow box). Right: Higher magnification view of the selected region. **(B–C)** Further magnified views of boxed regions in A, highlighting intermingled but non-overlapping distributions of *Vglut*1^+^ and *Vgat*^+^ cell populations. The thickness of the maximum intensity projection for the images is 2 μm. **(D)** Enrichment score of *Vglut1*/*Vgat*. Each dot represents the centroid of a cell. The panels show 32 μm axial projections of sub-volumes arranged from anterior to posterior (left to right). **(E)** Estimated subregion boundary (yellow line) based on molecular cell distribution for each Z section. **(F)** Alignment of the molecular boundary to the Allen CCFv3 anatomical atlas across four Z sections (Z1 to Z4). **(G)** Quantitative comparison between molecular and anatomical boundaries using MAE, AUC and Pearson correlation coefficient across Z1 to Z4. **(H)** Schematic illustrating the method for calculating boundary deviation. **(I)** Plot of boundary deviation along the dorsoventral (DV) axis for each Z section (Z1–Z4).

The comprehensive spatial distribution of different cell types enables the construction of a molecularly defined brain atlas (Zhang et al., 2023). The EASI-FISH technique enables simultaneous acquisition of spatial localization and transcriptional profiles of individual cells at single-cell resolution (Wang et al., 2021). By selecting representative marker genes (such as *Vglut2, Vgat, Otp*, and *Meis2*), and applying principal component analysis, the study revealed distinct spatial expression patterns of these genes within the lateral hypothalamic area (LHA). Leveraging high-throughput spatial transcriptomics in combination with machine learning approaches, the researchers achieved a systematic sub-regional delineation of the mouse LHA. Inspired by the above findings, this study observed a mutually exclusive distribution of *Vglut1*- and *Vgat*-labeled cell types near the boundary between the reticular nucleus of the thalamus (RT) and ventral part of the lateral geniculate complex (LGv) regions (Figures 5A–C). This distribution pattern closely aligns with the gradient direction and spatial positioning of the LGv-RT boundary as defined in the CCFv3 annotation.

To evaluate the differences between molecularly defined boundaries and those annotated in CCFv3, we first determined the spatial locations of *Vglut1*- and *Vgat*-labeled cells based on segmentation results. We then calculated the relative enrichment scores for each pair of cell types. Finally, axial projection heatmaps were generated for four sub-volumes, using the enrichment scores of individual cells. It is evident that *Vglut*1^+^ and *Vgat*^+^ cells located near the boundary exhibit enrichment scores within the range of (-1, 1), while the majority of cells outside the boundary show enrichment scores close to 1 or -1 (Figure 5D). Based on the sign of the enrichment score, cells within each sub-volume were classified into two groups, and a gaussian mixture model was applied to estimate the boundary separating these two distributions (Figure 5E). The local clustering of cells and the transitional characteristics at the boundary were clearly visualized through the distribution maps.

After brain atlas registration, the spatial coordinates of the two cell groups and their boundary curves from the four axial projection maps were mapped onto the brain atlas and compared with the annotated boundary curve on the left side of the RT region. The RT region boundary curve was manually annotated by domain experts using ilastik (Berg et al., 2019). To assess the differences between the two boundary curves, we compared them within each projection using the same start and end coordinates. The evaluation was performed using three metrics: mean absolute error (MAE), area under the curve (AUC), and pearson correlation coefficient (Figure 5F). Among the four axial projection sub-volumes, the boundary curves in sub-volume Z4 exhibited lower MAE and AUC values, indicating a higher degree of overlap between the two curves. Across all sub-volumes (Z1–Z4), the curves demonstrated strong correlations in their trajectories (Pearson correlation coefficients > 0.5), suggesting a high level of consistency in their overall patterns (Figure 5G).

To more precisely assess the differences between the two curves, we calculated the shortest distance from each point on the atlas-annotated boundary (Curve B) to the molecularly defined boundary (Curve A) along the normal direction (Figure 5H). This provides a quantitative measure of the spatial displacement between the two curves. Across the different axial projection maps, the overall trend indicates that the molecular boundary initially deviates from the atlas annotation but gradually converges toward it in the ventral direction. In the Z4 projection, the molecularly defined boundary shows the closest alignment with the atlas annotation along the dorsal-ventral (DV) axis compared to the other projections (Figure 5I).

## 4 Discussion

In this study, we successfully acquired high-resolution, thick-section image datasets of excitatory and inhibitory cell types using multi-FISH labeling combined with the high-speed volumetric imaging technique VISoR. As a critical step in cell type identification, accurate cell segmentation is essential for the analysis of neuronal cell-type image data. However, due to the heterogeneity in size and intensity among different signal types, relying solely on manual annotation incurs prohibitively high labor costs. To address this challenge, we propose VUSMamba, a self-supervised learning-based neural network capable of extracting generalizable features from unlabeled images. The model was then fine-tuned using a small set of annotated data via transfer learning. The resulting VUSMamba network demonstrates robust performance and consistently outperforms existing baseline models on multiple segmentation metrics. Moreover, thanks to the linear time complexity of the Mamba architecture, the model exhibits significant advantages in computational efficiency and hardware compatibility.

This study primarily focuses on achieving accurate cell segmentation in brain slices from multiple samples; however, it has not been yet extended to whole-brain datasets encompassing multiple cell types. Such datasets typically range from terabytes to petabytes in size and often include multimodal information—such as structural imaging, molecular markers, and functional activity—which imposes substantial demands on model's computational complexity. Efficient processing of these large-scale data requires not only optimized algorithm design but also the support of high-performance computing resources, such as GPU clusters. This study shows that segmentation errors may occur when cells are closely adjacent, due to inconsistencies in boundary annotations among different annotators, which in turn affect model fine-tuning. Furthermore, variations in imaging modalities (e.g., light-sheet microscopy, electron microscopy) and experimental conditions (e.g., tissue clearing protocols, staining techniques) can introduce distribution shifts in the data, potentially impairing model generalization. Conventional approaches often rely on dataset-specific fine-tuning, which increases computational cost and limits the scalability and universality of the model. The deep learning model proposed in this study also faces this challenge. Therefore, developing a unified deep learning framework capable of adapting across modalities and experimental conditions is essential for advancing large-scale, robust analysis of whole-brain imaging data (Li et al., 2023).

The neocortex and hippocampal regions of the mammalian brain play critical roles in higher-order neural functions such as perception, cognition, emotion, and learning. These two major brain areas are primarily composed of two main types of neurons: glutamatergic excitatory neurons and GABAergic inhibitory neurons, both of which have been central subjects in neuroscience research (Yao et al., 2021, 2023). Our analysis reveals that, across most brain regions, neurons predominantly express either glutamatergic or GABAergic marker genes, with single-gene-expressing neurons accounting for the majority. Notably, a significant proportion of neurons co-express excitatory and inhibitory gene markers (e.g., *Vglut1*-*Vgat*), suggesting molecular heterogeneity within these populations. These findings highlight the diversity in gene expression patterns among glutamatergic and GABAergic neurons and further support the hypothesis that functional interactions may exist between excitatory and inhibitory neuronal subtypes (Pelkey et al., 2020; Kim et al., 2022; Li et al., 2024).

The CCFv3 brain atlas is primarily based on cytoarchitectural features (e.g., Nissl staining) and a limited number of molecular markers, which may overlook heterogeneity at the functional or molecular level. Increasingly, the spatial distribution of molecularly defined cell types is being recognized as a key reference for delineating brain regions (Zeng and Sanes, 2017). However, this approach requires comprehensive mapping of a broader range of cell-type-specific spatial distribution patterns. Since this study focused on labeling only two neuronal populations, specifically glutamatergic and GABAergic cells identified by *Vglut1* and *Vgat* expression, it was not feasible to delineate subregions based on the distribution of multiple cell types. However, quantitative analysis still enabled the identification of a boundary within the RT region that effectively separates the spatial distribution of these two cell populations. This molecularly defined boundary showed a strong correlation with the boundary annotated in CCFv3, with better spatial alignment observed on the ventral side compared to the dorsal side.

In summary, this study presents an efficient and generalizable framework for high-resolution cell-type segmentation in thick brain slices, leveraging self-supervised learning to overcome challenges posed by signal heterogeneity and limited annotations. Although our current study focuses only on a subset of neuronal populations within thick brain slices, the results highlight the potential of integrating molecular labeling with advanced computational methods to uncover principles of brain spatial organization. Future extensions to whole-brain, multimodal datasets will further enhance our understanding of neuronal diversity and brain architecture.

## Data Availability

The Hoechst, *Vglut1*, and *Vgat* image data used in this study are publicly available at the following link: https://zenodo.org/records/15735899.
