# Supplementary material for: Self-supervised learning analysis of multi-FISH labeled cell-type map in thick brain slices
Source: Front Neurosci. 2025 Jul 7;19:1622950. doi: 10.3389/fnins.2025.1622950 (PMC12277362; doi:10.3389/fnins.2025.1622950)
Supplement: Supplementary file 1 [file Data_Sheet_1.PDF]

Supplementary Material

1 MICE

All animal experiments were approved by the Animal Care and Use Committee of the University of Science and Technology of China and conducted in accordance with institutional guidelines. Mice were group-housed (three to five per cage) under a 12-hour light/dark cycle, with controlled temperature and humidity, and had free access to food and water. Adult male C57BL/6 mice aged 8 to 12 weeks were used in the study.

2 HCR PROBE AND HAIRPIN DESIGN

Target gene mRNA sequences were obtained from the NCBI Gene database (`https://www.ncbi.nlm.nih.gov/gene`). Hybridization Chain Reaction (HCR) 3.0 probe pairs were designed using the OligoMiner tool (`http://oligominerapp.org/`) Beliveau et al. (2018); Passaro et al. (2020), and subsequently tagged with specific initiators (see Supporting Information). Fluorescent hairpins labeled with Alexa Fluor 488, ATTO 550, and Alexa Fluor 647 were utilized in the experiments Choi et al. (2018). Both the HCR probe pairs and fluorescent hairpins were synthesized by Sangon Biotech (Shanghai, China) and purified using UltraPAGE and High-Performance Liquid Chromatography (HPLC).

3 BRAIN SLICE RECONSTRUCTION

The reconstruction procedure Wang et al. (2019) consists of five main stages: (1) Based on the actual coordinates of the acquired images, they were first organized into columns. Full brain slices were then reconstructed from these columns using correction factors and accurate overlapping region coordinates. (2) The top and bottom surfaces of each contrast-enhanced brain section were estimated using linear regression and interpolation techniques. (3) A combination of rigid transformation and the B-spline algorithm Marstal et al. (2016) was applied to identify textures and boundaries on the facing surfaces of adjacent slices. (4) To avoid the accumulation and spread of misalignment errors, constraints were imposed on the displacement relationships and vectors between neighboring slices. (5) Finally, slice distortion in terms of size and shape was reduced through a moving least squares approach Schaefer et al. (2006).

4 TRANSFER LEARNING

Following the self-supervised training, we acquired a pre-trained model. This pre-trained model is then subjected to transfer learning on the annotated dataset, resulting in the development of a robust neural network capable of end-to-end volumetric multi-cell types segmentation. The environment for transfer learning of the network remained consistent with the one described in the previous section. The optimization function utilized AdamW with a learning rate of 1×10^{-4} and weight decay of 0.05. The network was trained for 50 epochs with a batch size of 2 in transfer learning. The combination of Dice loss and cross-entropy loss has been empirically demonstrated to enhance the robustness of training medical image segmentation models. Hence, we selected them as the loss functions for the transfer learning phase and assigned appropriate weights to each. They are defined as:

$$loss_{ce} = -\frac{1}{N}\sum_{n=1}^{N}\log\left(\frac{\exp\left(x\left(n, y\left(n\right)\right)\right)}{\sum_{c=1}^{C}\exp\left(x\left(n, c\right)\right)}\right) \tag{S1}$$

$$loss_{dice} = 1 - \frac{2\left|Y \cap \tilde{Y}\right|}{|Y| + \left|\tilde{Y}\right|} \tag{S2}$$

where $x(n, c)$ represents the score of class c for the n-th sample. N is the batch size, and C is the number of classes. $y(n)$ denotes the ground truth class index for the n-th sample (Eq. (S1)). In Eq. (S2), $\tilde{Y}$ is the ground truth of image segmentation, and Y is the segmentation result predicted by the neural network. The total loss function is defined as:

$$loss = \theta_1 loss_{ce} + \theta_2 loss_{dice} \tag{S3}$$

where θ_1 and θ_2 are hyperparameters.

5 ATLAS REGISTRATION

To observe the distribution of cell types throughout the brain slices, atlas registration was necessary. First, we used Elastix Klein et al. (2010) (`http://elastix.lumc.nl`) to register the brain slices to the Allen Brain Atlas CCFv3. The brain registration process involved downsampling the 3D images to $25 \times 25 \times 25$ μm^3 voxel size, using Adaptive stochastic gradient descent for non-rigid B-spline transformation, and Advanced mattes mutual Information as the similarity metric. Three image pyramids with variable resolutions were used for interpolation and resampling, and the transformation parameters determined the cell coordinates in the brain slices.

6 QUANTIFICATION ANALYSIS

Cell density. To fairly compare the distribution differences of specific cell types across different brain regions, we used density as a measure of their regional distribution. Specifically, we first counted the number of specific cell types in a given brain region, then calculated the volume of that region based on the mouse brain atlas CCFv3. Finally, the density of the cell type in the region was defined as:

$$\text{Density} = \frac{\text{count}}{\text{volume}_{\text{region}}} \tag{S4}$$

where count referred to the number of specific cell types in the region, and $\text{volume}_{\text{region}}$ was the volume (mm^3) of the region.

Enrichment scores. The enrichment score was calculated by querying the number of occurrences of each neuronal subtype (c_i, d_i) within a radius r centered at the spatial location of each neuron. The formula is defined as follows:

$$S_i = \frac{c_i - d_i}{c_i + d_i} \tag{S5}$$

REFERENCES

Beliveau, B. J., Kishi, J. Y., Nir, G., Sasaki, H. M., Saka, S. K., Nguyen, S. C., et al. (2018). Oligominer provides a rapid, flexible environment for the design of genome-scale oligonucleotide in situ hybridization probes. *Proceedings of the National Academy of Sciences* 115, E2183–E2192. doi:10.1073/pnas.1714530115

Choi, H. M. T., Schwarzkopf, M., Fornace, M. E., Acharya, A., Artavanis, G., Stegmaier, J., et al. (2018). Third-generation *in situ* hybridization chain reaction: Multiplexed, quantitative, sensitive, versatile, robust. *Development* 145, dev165753. doi:10.1242/dev.165753

Klein, S., Staring, M., Murphy, K., Viergever, M., and Pluim, J. (2010). Elastix: A toolbox for intensity-based medical image registration. *IEEE Transactions on Medical Imaging* 29, 196–205. doi:10.1109/TMI.2009.2035616

Marstal, K., Berendsen, F., Staring, M., and Klein, S. (2016). Simpleelastix: A user-friendly, multi-lingual library for medical image registration. In *2016 IEEE Conference on Computer Vision and Pattern Recognition Workshops (CVPRW)* (Las Vegas, NV, USA: IEEE), 574–582. doi:10.1109/CVPRW.2016.78

Passaro, M., Martinovic, M., Bevilacqua, V., Hershberg, E. A., Rossetti, G., Beliveau, B. J., et al. (2020). Oligominerapp: A web-server application for the design of genome-scale oligonucleotide in situ hybridization probes through the flexible oligominer environment. *Nucleic Acids Research* 48, W332–W339. doi:10.1093/nar/gkaa251

Schaefer, S., McPhail, T., and Warren, J. (2006). Image deformation using moving least squares. In *ACM SIGGRAPH 2006 Papers* (New York, NY, USA: Association for Computing Machinery), vol. 25 of *SIGGRAPH '06*, 533–540. doi:10.1145/1179352.1141920

Wang, H., Zhu, Q., Ding, L., Shen, Y., Yang, C.-Y., Xu, F., et al. (2019). Scalable volumetric imaging for ultrahigh-speed brain mapping at synaptic resolution. *National Science Review* 6, 982–992. doi:10.1093/nsr/nwz053

7 SUPPLEMENTARY TABLE

Table S1. Information about datasets

	Image Size	No. Images	Storage Size	Physical Resolution
Self-training datasets	64 x 256 x 256	13736	214GB	2 μm^3/voxel
Transfer learning datasets	64 x 256 x 256	640	20GB	2 μm^3/voxel
Test datasets	64 x 256 x 256	960	7.50GB	2 μm^3/voxel

Table S2. An ablation study was conducted to assess the effectiveness of each objective function in the proposed pre-training loss through fine-tuning experiments on the Hoechst image dataset.

Loss function	Dice	Jc	Sst
l_{rot}	0.8763	0.8178	0.8489
$l_{contrast}$	0.8835	0.8212	0.8533
$l_{recovery}$	0.8891	0.8285	0.8579
$l_{rot} + l_{contrast}$	0.8913	0.8307	0.8614
$l_{rot} + l_{recovery}$	0.8938	0.8326	0.8635
$l_{rot} + l_{contrast} + l_{recovery}$	**0.8961**	**0.8339**	**0.8694**

Table S3. Comparative inference performance of VUSMamba and advanced baseline models

Model	#param	FLOPs	Inference time	Memory
VUSMamba	41.50M	13.82G	$\sim$ 7ms	$\sim$ 6.8G
3D-HSFormer	41.57M	101.88G	$\sim$ 9ms	$\sim$ 8.1G
3D-UNet	9.22M	615.06G	$\sim$ 10ms	$\sim$ 4.8G
CP-Net	11.40M	2.6T	$\sim$ 13ms	$\sim$ 5.7G
SwinUNETR	68.54M	452.84G	$\sim$ 11ms	$\sim$ 7.3G

8 SUPPLEMENTARY VIDEO

Supplementary video 1. The spatial distribution of HCR FISH-labeled glutamatergic (*Vglut1*$^+$) and GABAergic (*Vgat*$^+$) neurons in mouse brain slice.
Supplementary video 2. Visualization of *Vglut1*$^+$ and *Vgat*$^+$ neuron segmentation results using VUSMamba.

9 SUPPLEMENTARY FIGURE

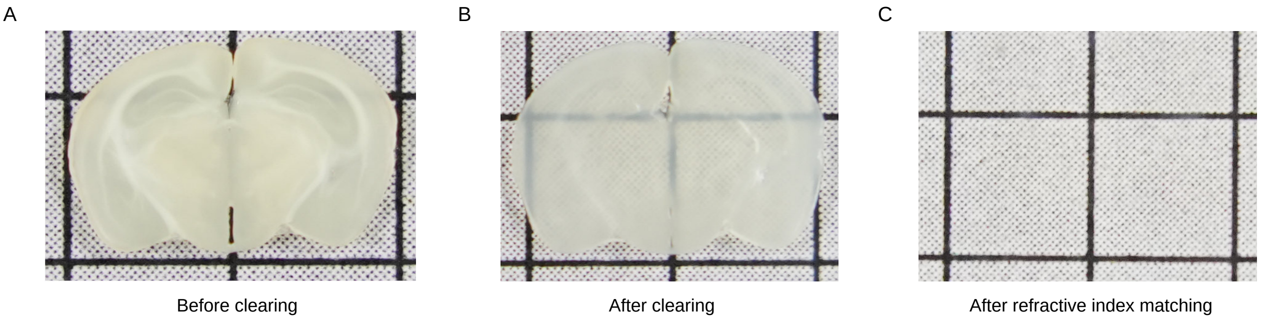

Figure S1: Tissue transparency at different stages of the clearing process. (A) Brain slice before clearing. (B) Brain slice after 4% SDS solution clearing. (C) Brain slice after refractive index matching. The blue markings indicate the position of the brain slice after refractive index matching. The black grid lines are spaced 5 mm apart in both length and width.

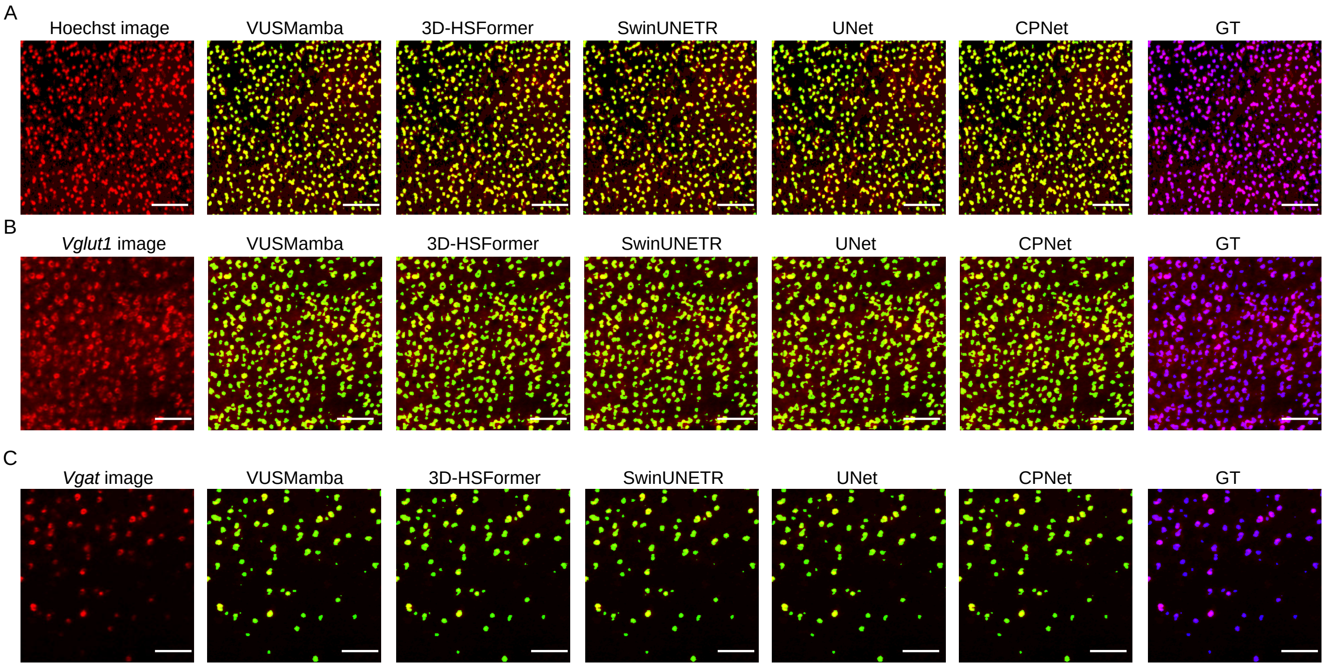

Figure S2: Comparison of cell segmentation performance across different models on multi-FISH labeled brain images. (A-C) Representative segmentation results of Hoechst (A), *Vglut1* (B), and *Vgat* (C) labeled brain sections using five different models: VUSMamba, 3D-HSFormer, SwinUNETR, UNet, and CPNet. The GT annotations are shown in the rightmost column for each row. Each model's prediction is overlaid on the original input image to visualize segmentation accuracy. The thickness of the maximum intensity projection for the images is 2 μm. Scale bars: 40 μm.

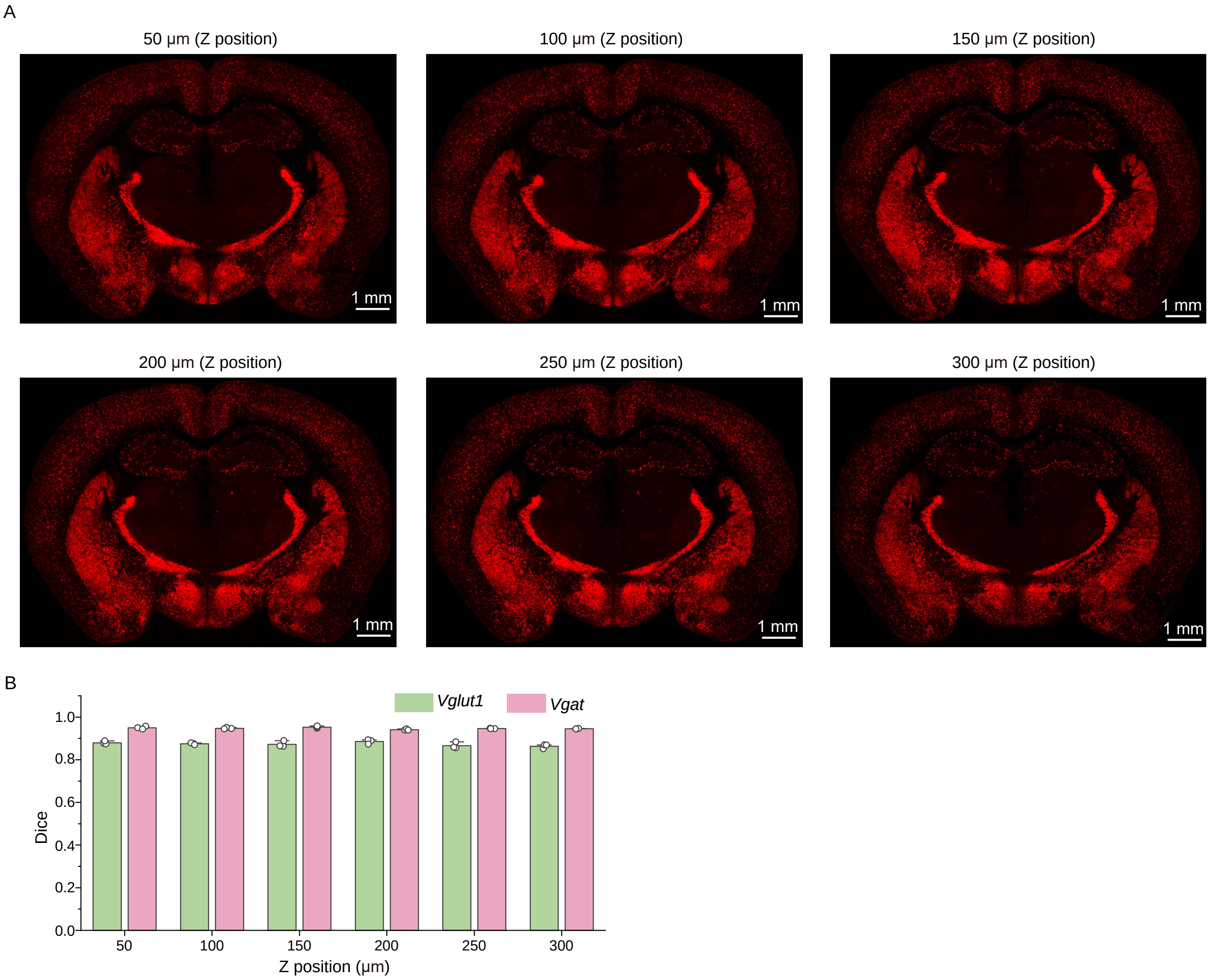

Figure S3: Evaluation of multi-FISH signal consistency and VUSMamba segmentation performance across z positions in the mouse brain. (A) Representative coronal brain sections imaged at six different z positions, showing FISH labeling of *Vgat* gene expression. The thickness of the maximum intensity projection for the images is 2 µm. (B) Quantification of segmentation consistency using the Dice coefficient for *Vglut1* and *Vgat* across the six z positions.

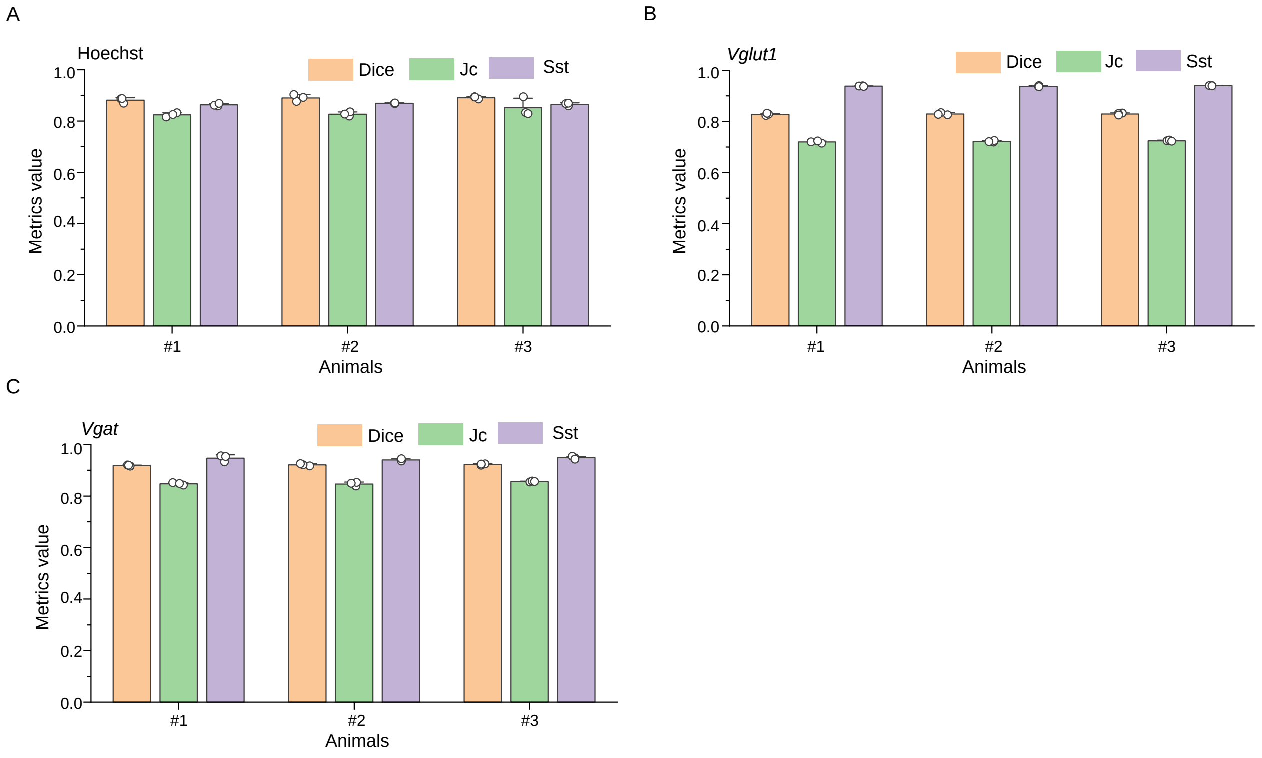

Figure S4: VUSMamba segmentation performance across multiple biological replicates. (A-C) Quantitative evaluation of VUSMamba's segmentation performance on Hoechst (A), *Vglut1* (B), and *Vgat* (C) signals from three adult mice. For each animal, segmentation was assessed on three consecutive brain slices (n=3) using Dice, Jc, and Sst.
