# Supplementary figures and images for: Self-supervised learning analysis of multi-FISH labeled cell-type map in thick brain slices
Source: Front Neurosci. 2025 Jul 7;19:1622950. doi: 10.3389/fnins.2025.1622950 (PMC12277362; doi:10.3389/fnins.2025.1622950)

A

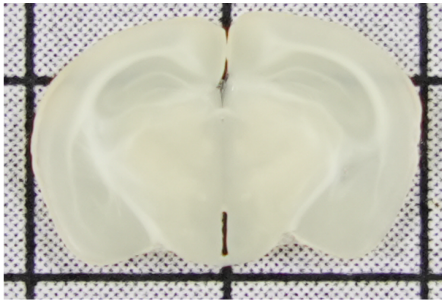

Before clearing

B

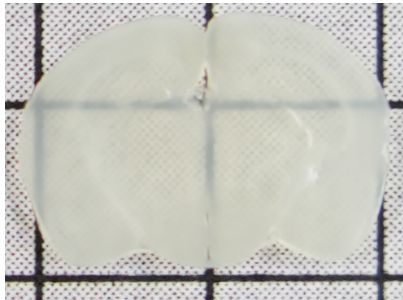

After clearing

C

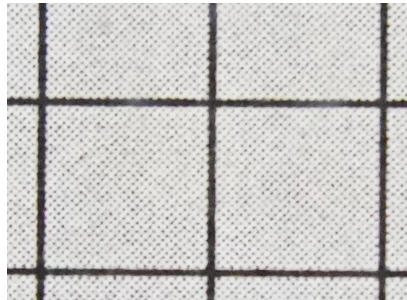

After refractive index matching

Supplement: Supplementary file 2 [file Image_1.PDF]

A

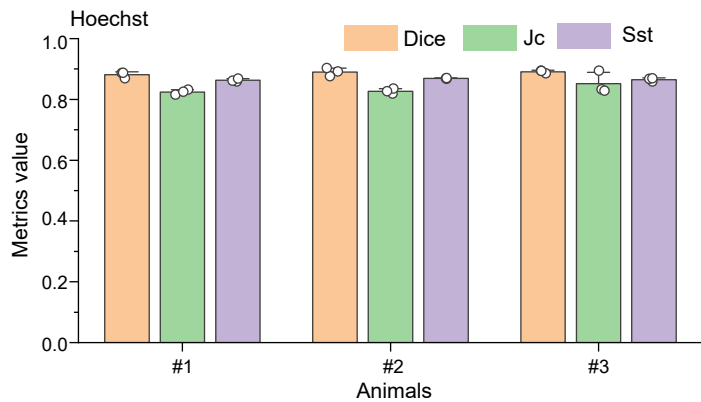

B

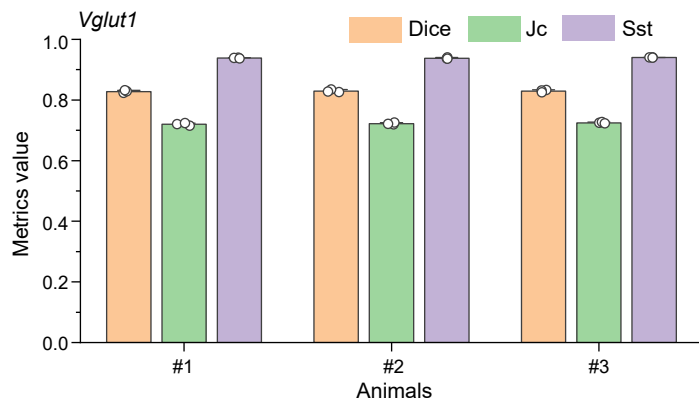

C

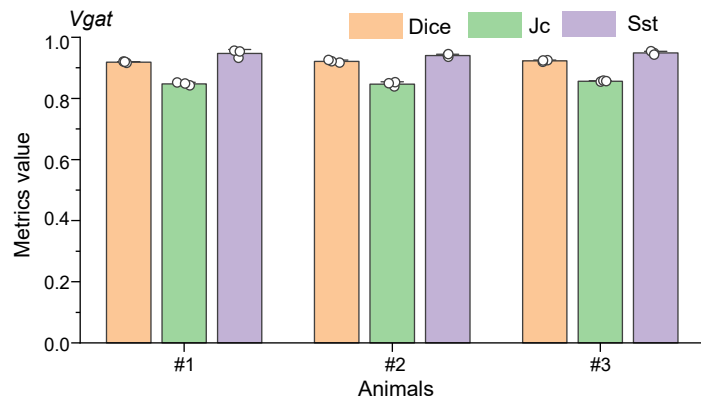

Supplement: Supplementary file 5 [file Image_4.PDF]
