# Supplementary material for: Self-supervised learning analysis of multi-FISH labeled cell-type map in thick brain slices
Source: Front Neurosci. 2025 Jul 7;19:1622950. doi: 10.3389/fnins.2025.1622950 (PMC12277362; doi:10.3389/fnins.2025.1622950)
Supplement: Supplementary file 3 [file Image_2.PDF]

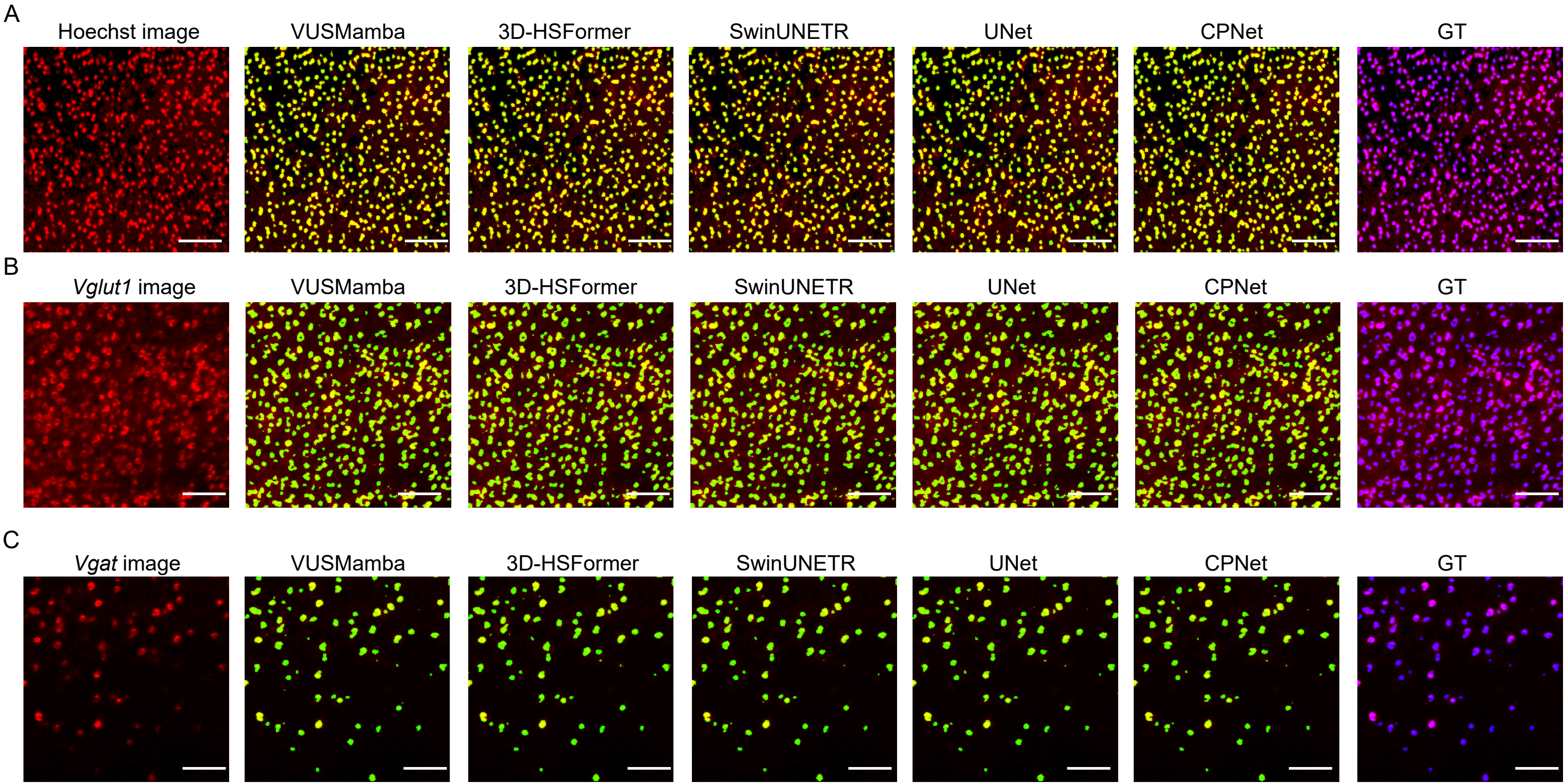

A
Hoechst image
VUSMamba
3D-HSFormer
SwinUNETR
UNet
CPNet
GT
B
Vglut1 image
VUSMamba
3D-HSFormer
SwinUNETR
UNet
CPNet
GT
C
Vgat image
VUSMamba
3D-HSFormer
SwinUNETR
UNet
CPNet
GT
