# Supplementary material for: Self-supervised learning analysis of multi-FISH labeled cell-type map in thick brain slices
Source: Front Neurosci. 2025 Jul 7;19:1622950. doi: 10.3389/fnins.2025.1622950 (PMC12277362; doi:10.3389/fnins.2025.1622950)
Supplement: Supplementary file 4 [file Image_3.PDF]

A

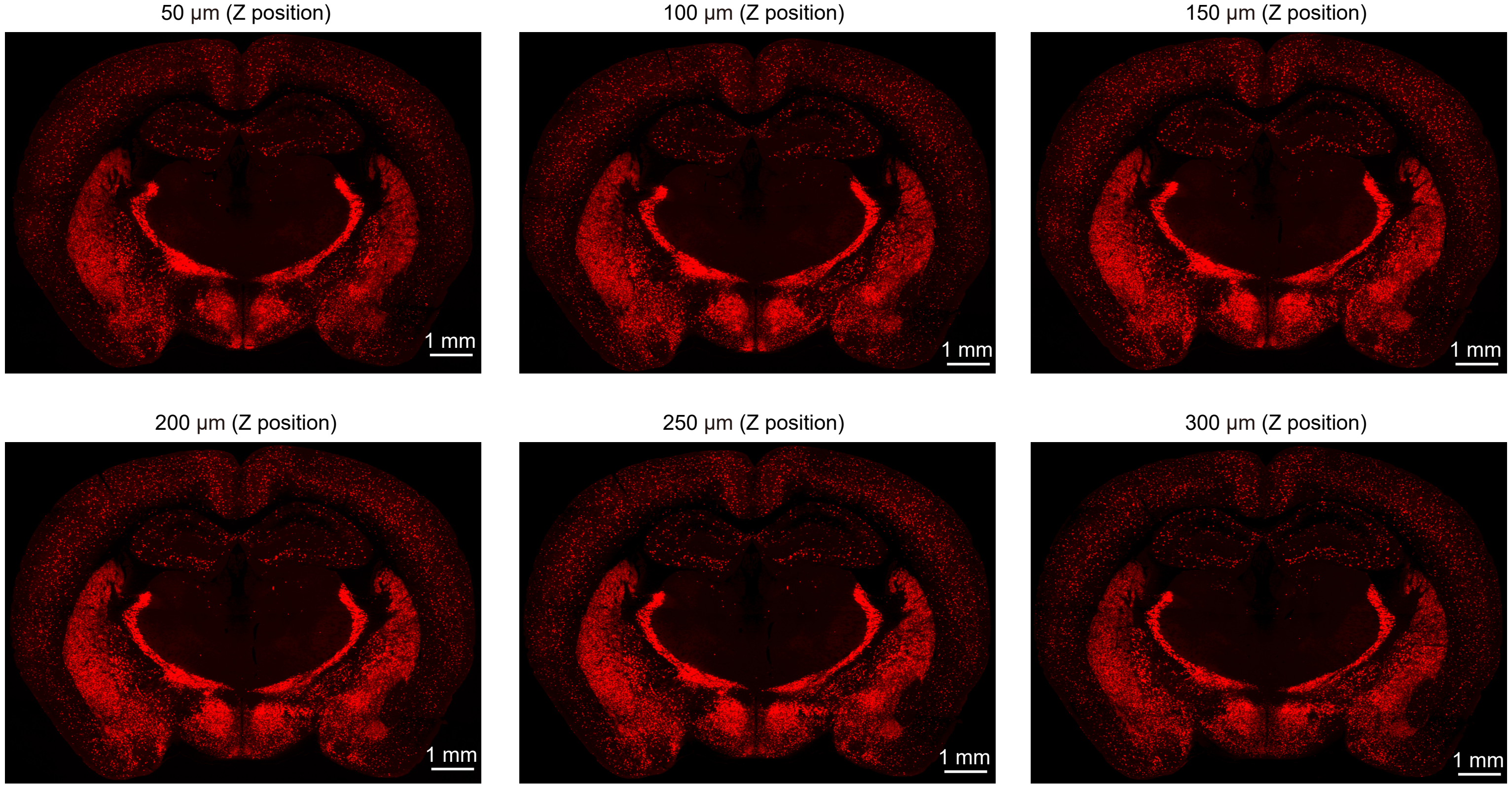
50 μm (Z position)
100 μm (Z position)
150 μm (Z position)
200 μm (Z position)
250 μm (Z position)
300 μm (Z position)
1 mm

B

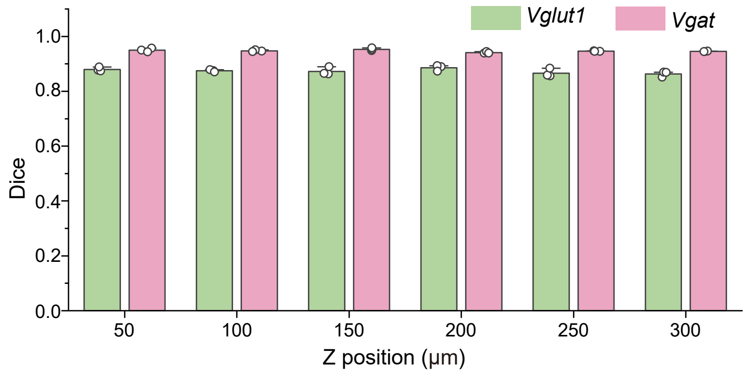
Vglut1
Vgat
Dice
0.0
0.2
0.4
0.6
0.8
1.0
50
100
150
200
250
300
Z position (μm)
